# Price premiums for meat products with sustainability labels

**DOI:** 10.1371/journal.pone.0353190

**Published:** 2026-08-12

**Authors:** Maria Berikou, Jinho Jung, Danielle J. Ufer, Nicole O. Widmar, Jayson L. Lusk

**Affiliations:** 1 Purdue University, Department of Agricultural Economics, West Lafayette, Indiana; 2 Pohang University of Science and Technology (POSTECH), Division of Humanities and Social Sciences, Pohang, Gyungbuk, Republic of Korea; 3 (Former) US Department of Agriculture, Economic Research Service, Independence Ave, SW, Washington DC, United States of America; 4 Purdue University, Department of Agricultural Economics, West Lafayette, Indiana; 5 Oklahoma State University, Division of Agricultural Sciences & Natural Resources, Stillwater, Oklahoma; Newcastle University, UNITED KINGDOM OF GREAT BRITAIN AND NORTHERN IRELAND

## Abstract

With consumers’ growing demand for products labeled as having been produced without practices or substances, agricultural producers are given the chance to distinguish their products through eco-labels and other claims. During the last few decades, a number of food labels have been developed to communicate sustainability information, with consumers interested in the carbon footprint and practices of agriculture in recent years. This study investigates the retail premiums for such sustainability labels as greenhouse gas (GHG) emission-reducing practices and other potentially relevant sustainability labels on meat products, including organic, grass-fed, gluten-free, and whether genetically modified organisms were used in production. Prices and labeling information about beef, pork, and chicken products in selected stores from 48 states were collected via web-scraping and investigated for sustainability labels. Market-observed premiums for such labels as reduced-GHG and sustainable practices were investigated alongside impacts of geography on product prices. Our results showed significant premiums for almost all sustainability claims investigated. Among the claims studied, Organic and Grass-fed labels consistently carried positive premiums across all three meat types, with pork products generally exhibiting the highest premiums and chicken products the lowest.

## 1. Introduction

Conventional agricultural systems employ a variety of practices that a growing contingent of consumers would prefer to be absent from food production. In avoiding these practices, consumers are often seeking to reduce the negative impacts of agricultural production, whether they be associated with public or personal health, environmental sustainability, or ethics [1, 2]. Among the outcomes of agricultural production that consumers are increasingly interested in avoiding are excess greenhouse gas (GHG) emissions, especially those associated with livestock production [3]. Online and social media data studied in the U.S. suggests that the public is increasingly aware of cattle production’s role in GHG emissions [1]. Conventional agricultural production has been found to result in high emissions, with modern food systems accounting for more than one-third of anthropogenic GHG emissions [4]. As consumers and other stakeholders value the sustainability of agricultural production, especially livestock production, an opportunity thus arises for market offerings that meet demand for lower emissions and more sustainable environmental footprints.

It is becoming common for consumers to incorporate perceived environmental impacts, in addition to other production process attributes, into food purchasing decisions [5]. Sustainability labels are one way that producers can capitalize on this opportunity. Sustainability labels can both inform consumers about the environmental impacts of their food choices, potentially encouraging consumption of foods with lower emissions [6–10,11] and meet consumer demand for products that align with their sustainability preferences [12,13]. With respect to carbon, a novel sustainability label beginning to enter food markets is the “carbon footprint” (CF) label which attempts to demonstrate the total impact of a product as communicated through standardized carbon dioxide emissions measurements [14]. Evidence from several studies indicates that consumers value foods produced with sustainability practices and are willing to pay substantial premiums to purchase such products [see, for example, 2,15–18]. The majority of these studies, however, have been conducted outside of the United States and primarily concentrate on consumer values for products which might not even be commercially available. This raises the question of the extent to which such products have become an available option for the U.S. consumers in the grocery aisle, as well as the degree to which retail market premiums actually reward products with sustainability labels.

Although the market for sustainability labels in the U.S. retail animal products has not been studied extensively, research on other labels of a similar nature is well-represented in the literature and may offer some insights. Just as the CF label represents less GHG emissions and environmental impacts of conventional agricultural production, several of the most common specialty food labels in the U.S. advertise the absence of production practices or substances which consumers might find undesirable [19]. This is especially so for meat products, where sustainability labels may include organic, less greenhouse gas, no genetically modified organisms (non-GMO), raised without antibiotics, no added hormones, gluten-free, and grass-fed, among many others. Some labels placed on products, while true, are somewhat confusing in the sense that it labels something that was true anyway. For example, meat is gluten-free unless something is added to it. Thus, labeling meat as gluten-free is truthful, yet it isn’t specific to that product but rather all meat whether labeled or not. Many of these practices are also perceived as influencing the sustainability of a food’s production. One of the best studied sustainability claims in meat is the organic claim for beef. Schulz et al. [20] reported a significant price premium of $2.98 per pound for organic beef steaks. Relative premiums for organic beef reported in the literature have ranged from 40% to 115% [21–23]. High premiums are not just observed for organic beef but have also been observed for other meats like pork and chicken [23]. Additional retail premiums have also been observed for other sustainability claims, both on their own and when concurrent with an organic claim [21]. Grass-fed labels on beef have been found to carry premiums of 34% to as high as 193%, depending on the cut of beef [24,25]. Consumers with strong preferences for one absence claim, such as no added hormones/antibiotics, may also be more likely to pay a premium for other claims, like grass-fed [26]. As with organic, premiums for sustainability labels can extend to other meats, too. For example, Nilsson et al. [27] found some consumers were willing to pay a premium of between 6 and 19% for pork chops certified to meet strict environmental, animal welfare, and antibiotic-use standards. Along with the increased perceived value to environment, health, animal welfare, etc. that consumers derive from them, products with various sustainability labels and claims usually carry premiums due to the added costs of production practices and/or manufacturing procedures of such certified food products [28,29]. These studies together demonstrate that there exists both perceived value of such claims in meat products, as well as real economic compensation for these production practices as exhibited by the retail premiums observed in the market. Though the literature has explored the perceived value of sustainability labels on meat, through willingness to pay studies, it remains to be seen what the real retail market status is for sustainability labels on meat.

Sustainability labels are an emerging category of labels for animal products in the United States. These labels have the potential to offer several benefits to the U.S. consumer, but their retail market status in terms of both availability and value are not well understood. While current research sets a market context for these labels, it remains unclear the true extent to which sustainability labels are present in the retail meat market. Furthermore, as retail premiums for other labels have exhibited considerable heterogeneity and premiums function as the core market incentive for driving specialty market offerings, it is critical to understand the current retail premiums for sustainability labels. In this study we define sustainability labels to indicate such claims as *organic*, *less greenhouse gas*, *grass-fed*, *gluten-free*, and whether genetically modified organisms were used in production. To investigate the current market status of sustainability labels in U.S. retail meats, we employ a novel dataset of retail meat product prices and label information compiled using web-scraping techniques across 342 Zip codes in 20 major U.S. grocery store chains. We estimate hedonic pricing models for sustainability claims on retail beef, pork, and chicken products. These models include several of the most common labels for meat products in order to contextualize the identified market premiums for sustainability labels and further contribute to the literature on food labels and specialty trait premiums in U.S. retail animal products.

## 2. Data and methodologies

### 2.1. Data collection – web scraping

Web scraping, also referred to as web extraction or web harvesting, is a method of extracting information from the Internet and storing it in a database. By using web scraping techniques, data in the online space can be extracted from websites using HTTP (Hypertext Transfer Protocol) or a web browser [30]. It can serve many different analytical ends such as monitoring changes in prices of products or stock, collecting product reviews, gathering real estate listings, or monitoring the weather [30, 31].

In recent years, with the growth in online retailing, it has become efficient to collect web data about the prices of goods offered by supermarkets and other retailers. Web scraping has already been used for many kinds of research. As part of the Billion Price Project at MIT (2008), Cavallo and Rigobon [32] created an inflation series based on web-scraped online micro price data. Jung et al. [33] used web scraping in order to analyze online grocery markets in South Korea. Kim et al. [34] web-scraped restaurant data from a delivery application to investigate the geographical market structure of a food service industry in South Korea. Aravind and Sweetlin [35] employed web scraping to interpret nutritional food fact labels and details. Huang et al. [36] used a web scraping technique to look into the proportion of UK menu items exceeding recommended energy and nutrient intake values. Juszczak [37] used price data scraped from one of Poland’s largest online stores to investigate the use of web-scraped data and compare known price index formulas that have been applied in the web-scraping case and validate their sensitivity to the type of data filter used.

Data collection with web scraping can be divided into two consecutive steps: obtaining web resources and processing the web-scraped data for extracting needed information [30]. First, the entire web scraping procedure begins with selecting websites from which the data will be scraped, collecting product information from the chosen websites, and processing the scraped raw data into a structured format for extracting the specific information needed [38].

Processing the collected dataset is an important step when it comes to web-scraped data because, according to De Boe [39] and Eberebdu [40], 85% of the big data recently generated in online spaces is unstructured, which means data does not have a pre-defined manner of organization. Unstructured data is usually qualitative and text-heavy with irregularities and ambiguities, making it difficult to find regular patterns from the raw data using traditional methods of data analysis. For example, individual food products have specific product information provided using terms such as organic, grass-fed, and so on, which may be located in differing places on packaging and/or labels. As a result, the second phase of the web scraping process is for the raw scraped data to be transformed to produce a structured dataset through text mining analysis.

This study uses a novel dataset collected and maintained by the Center for Food Demand Analysis and Sustainability (CFDAS) at Purdue University. Product information was collected on a daily basis from the websites of 20 grocery chains with online presence (Table 1), representing 342 Zip codes in 47 U.S. states plus Washington DC (Table 2).

**Table 1:** Retailers in the dataset.

Owning firms	Grocery Stores
Albertsons	Albertsons, Safeway, ACME, Star Market, Shaw’s, Jewel Osco
Kroger	Kroger, Ralphs,’ Food 4 Less, Foods Co., Fred Meyers, QFC, Smith’s, City Market, King Soopers, Dillons, Gerbes, Fry’s, Payless
Meijer	Meijer

**Table 2:** Regions with the States.

West		Southern Plains		Midwest		Delta		Southeast		Northeast	
California	CA	Texas	TX	Illinois	IL	Arkansas	AR	Kentucky	KY	Maine	ME
Oregon	OR	Oklahoma	OK	Indiana	IN	Louisiana	LA	North Carolina	NC	Vermont	VT
Washington	WA			Iowa	IA	Mississippi	MS	Tennessee	TN	New York	NY
Montana	MT			Ohio	OH			Virginia	VA	Massachusetts	MA
Idaho	ID			Missouri	MO			West Virginia	WV	Connecticut	CT
Wyoming	WY			Michigan	MI			Alabama	AL	Rhode Island	RI
Nevada	NV			Minnesota	MN			Georgia	GA	New Hampshire	NH
Utah	UT			Wisconsin	WI			South Carolina	SC	New Jersey	NJ
Colorado	CO			North Dakota	ND			Florida	FL	Pennsylvania	PA
Arizona	AZ			South Dakota	SD					Delaware	DE
New Mexico	NM			Nebraska	NE					Maryland	MD
Alaska	AK			Kansas	KS					Washington, DC	DC
Hawaii	HI										

Supermarkets and grocery stores were selected in states which have online grocery websites, offer delivery services, and do not prohibit automated data collection, data mining, scraping, or collecting in their website terms and conditions of use. Besides, data collected is not utilized for making profits, but for research purposes. In the first phase of data collection, product information from the listing pages such as prices, product titles, weights, and uniform resource locators (URLs) were web-scraped and organized into individual columns in the dataset. Web scraping of product information from a listing page included collecting the price displayed, the product name (such as “80/20 ground beef tray” or “boneless beef stew”) and the product weight as displayed, such as “1 lb” or “2 lb.” However, product listing pages do not provide such information as labels nor product details. Each product must be clicked upon to get the product details and labels, which takes 2–3 extra seconds to load the product page and web-scraped such information. Considering there are as many products as thousands meat items at one zip code in one grocery store which aggregate up to a few hundred thousand products a day, it is not technically viable to web-scrape detailed products’ pages daily basis. Suppose there are a half millions of products a day across 342 Zip codes and 20 store chains for total, each of which takes 3 seconds to load and web-scrape one detailed product page upon a single click. It would take 416 hours to web-scrape all of them, which is not possible to complete in a single day. This study measures price premium of the labeled products in comparison to non-labels products, which makes cross-sectional data adequate to conduct such price premium estimation. Web-scraping labels and detailed product information while minimizing time of web-scraping process, detailed pages of all products in the datasets collected weekly over a month of September 2022 are web-scraped in the second phase.

In the second phase of data collection, the URLs of individual products based on the CFDAS’s scraped dataset were collected for beef, pork, and poultry for every Thursday in September 2022 given that grocery stores usually update prices and promotions once a week. The selected URLs were then looped to load individual products’ detailed webpages and, using the web scraping technique, labels and descriptions were collected to get more detailed characteristics of individual products such as growing methods, eco-labels, or other claims. Titles and labels of food products registered in the online grocery stores also contain detailed information such as package prices, weight (used to estimate unit price in $/pound), product claims, packaging, and details about processes used in production of the products (i.e., smoked, trimmed, cooked), while they are unstructured and vary across products. Merging the four weekly datasets of September 2022, the whole dataset (S1 Data) is made up of a total of 753,368 meat observations collected, of which 425,761 (56.5%) were collected for beef, 196,450 (26.1%) for chicken, and 131,157 (17.4%) for pork products.

Following the compilation of unstructured titles and labels, text mining was applied to extract detailed information and define structured variables for statistical analysis such as weight, product claim, detailed methods of growing or processing of products. With respect to claims, the variables generated from the mined text included grass-feeding/-fed, organic, whether the product is non-GMO, less greenhouse gas, cut style (e.g., ground versus steak for beef), and final product designations such as sausages.

### 2.2. Methodology

This study builds a hedonic price model with the goal of exploring the impact of labels on the price of agricultural products, specifically meats. The model is a log-linear model and price premiums were calculated following Halvorsen and Palmquist [41]. The log-linear form for the hedonic price equation is as follows:


$$\begin{array}{c} \text{ln}\left(Y\right)=\beta_{\text{0}}+X\beta +\varepsilon \end{array}$$

where Y is a column vector with unit prices of individual products ($ per pound), X is a matrix with explanatory variables including product claims, regions, store chain ownership, and meat cuts. ***ε*** is a column vector of error terms.

Premiums for product claims on beef, chicken, and pork were estimated individually for a total of three models. The prices for beef, chicken, and pork products depend on their attributes. To quantify an attribute’s premium or discount, we regressed the natural logarithm of price of products on binary variables, which take values between 0 and 1. The empirical model can be described more specifically with explanatory variables as follows:


$$\begin{array}{cc} Price=\; & \beta_{\text{0}}+Promotion{\cdot \beta }_{Promtion}+ProductClaims{\cdot \beta }_{ProductClaims}\\ & \;\;\;+Regions{\cdot \beta }_{Regions}+StoreChains{\cdot \beta }_{StoreChains}\\ & \;\;\;+MeatCuts{\cdot \beta }_{MeatCuts}+\varepsilon \end{array}$$

where Promotion is a vector of an indicator whether the product was being offered at full price or on a promotion, ProductClaims is a vector of claim variables indicating if a product is organic, grass-fed, gluten-free, and non-GMO in addition to less greenhouse gas with a conventional product without such claims as a base product*.* The inclusion of these variables both controls for the various traits that might occur alongside the sustainability claim, but also contextualizes the sustainability label in the market, allowing for comparisons of implicit prices with those of more established specialty and sustainability claims. The model also contains dummy variables for region (Regions) with Midwest as a base region, major grocery firm (StoreChains**)**, and meat cut or style (MeatCuts). Regions is a vector of variables indicating whether products are collected from Zip codes in *Delta, Northeast, Southeast, Southern Plains*, or *West* with *Midwest* as a base region. Definitions of such regions are illustrated in Table 1 above. StoreChains is a vector of which store chain products are on sale, *Albertsons* or *Meijer,* with *Kroger* as a base chain. Controlling for meat cut or style is essential, given meat prices and specialty premiums have been widely demonstrated to be product category-dependent [22,25,Winterstein & Habisch, 2020, 29,42,43]. For example, Dennis [44] found that the premium for organic beef varied from $2.96 per pound for a boneless top sirloin steak to $6.47 per pound for a boneless New York strip steak. Discrepancies in premiums were also observed in chicken, with bone-in and boneless products experiencing different organic premiums [44]. A variable of *Meat cut* addresses differently for beef, chicken, and port individually in each of the three models because each meat has different kinds of cutting process.

With cross-sectional data, there is a risk of heteroskedasticity which can result in inefficiency. A Breusch–Pagan–Godfrey (BPG) test for heteroskedasticity was performed, which is more sensitive to heteroscedasticity when analyzing a large dataset [45], to identify heteroscedasticity. The BPG test did not identify heteroscedasticity in the models. In addition, the robust standard error was also applied to make sure homoscedasticity in the models of this study due to its larger dataset suggested by Baum [46], King and Roberts [47], and Long and Ervin [48]. It is found that the models are not sensitive to potential violations of the homoskedasticity assumption.

Following estimation, the coefficients can be used to calculate implicit prices or premiums for the associated product characteristics. For continuous variables, the regression coefficient, if multiplied by 100, represents the percentage change in the product price given a unit change in the explanatory variable. But the percentage effect of a change of a dummy explanatory variable from 0 to 1 equals 100 *[exp ($B_{\text{1}}$) – 1], where $B_{\text{1}}$ is the relevant coefficient [41,49,50]. As [41], as cited in [50] have shown the coefficient of a dummy variable in semilogarithmic regression equals:


$$\begin{array}{c} B_{\text{1}}=ln\frac{\text{1}+\hat{Y_{\text{1}}}-\hat{Y_{ref}}}{\hat{Y_{ref}}}\; \end{array}$$

where ${\hat{Y}}_{\text{1}}$, is the predicted group value of $\hat{Y}$ for the group coded 1 and ${\hat{Y}}_{ref}$ is the predicted value of Y for the reference group. In order to find the percentage effect of the dummy variable on Y, it is necessary to use the inverse of the logarithmic function [50].

## 3. Results

### 3.1. Summary statistics: market availability

Table 3 illustrates the summary statistics of labels or product claims, regions, and grocery chains, and the proportion for dummy variables. *Organic* products (Organic = 1) accounted for 1.79% of observations, 1.56% were labeled as *Grass-fed*, 8.16% were denoted *Gluten-free*, 0.58% were *Non-GMO*, 0.13% were labeled as having produced *Less greenhouse gas*, 54.75% were advertised at a discount (*Promotion*), 18.87% of States belonged to the Midwest region, 2.56% belonged to the Delta region, 25.95% belonged to the Northeast region, 7.28% belonged to the Southeast region, 5.31% belonged to the Southern Plains region and finally 40.02% belonged to the West region. The owning firm Albertsons had 63.47% of observations, and Kroger and Meijer had 27.95% and 8.58%, respectively.

**Table 3:** Summary statistics.

Variable/Label	Value	Frequency (N = 1,527,373)	Proportion
*Product Claims*			
Organic	1 = Organic 0 = Not Organic	27,330 1,500,043	1.79%
Grass-fed	1 = Grass-Fed 0 = Not Grass-Fed	23,832 1,503,541	1.56%
Gluten-free	1 = Gluten-Free 0 = Not Gluten-Free	124,609 1,402,764	8.16%
Non-GMO	1 = Non-GMO 0 = Not Non-GMO	8,928 1,518,445	0.58%
Less greenhouse gas	1 = Less Greenhouse gas 0 = Not Less Greenhouse gas	2,027 1,525,346	0.13%
Promotion	1 = Yes 0 = No	836,313 691,060	54.75%
*Regions*			
Midwest	1 = Midwest 0 = No Midwest	288,188 1,239,185	18.87%
Delta	1 = Delta 0 = No Delta	39,171 1,488,202	2.56%
Northeast	1 = Northeast 0 = Not Northeast	396,429 1,130,944	25.95%
Southeast	1 = Southeast 0 = No Southeast	111,216 1,416,157	7.28%
Southern Plains	1 = Southern Plain 0 = No Southern Plain	81,104 1,446,269	5.31%
West	1 = West 0 = No West	611,265 916,108	40.02%
*Store Chains*			
Albertsons	1 = Albertsons 0 = No Albertsons	969,485 557,888	63.47%
Kroger	1 = Kroger 0 = No Kroger	426,830 1,100,543	27.95%
Meijer	1 = Meijer 0 = No Meijer	131,058 1,396,315	8.58%

A comparison of mean prices across product categories for beef, chicken, and pork with and without labels is also shown in Table 4. The mean prices for beef, regarding the *Organic, Grass-fed, Gluten-free, Non-GMO, Less greenhouse gas* labels were: $15.93 per pound, $14.34 per pound, $7.97 per pound, $14.10 per pound, and $12.36 per pound, respectively. The mean prices for chicken, for the characteristics of *Organic, Grass-fed, Gluten-free, Non-GMO, Less greenhouse gas* were: $8.31 per pound, $10.19 per pound, $8.86 per pound, $8.94 per pound, and $11.86 per pound, respectively. The mean prices for pork for *Organic, Grass-fed, Gluten-free, Non-GMO* traits were $11.51 per pound, $9.32 per pound, $7.08 per pound, and $12.67 per pound, respectively.

**Table 4:** Summary Statistics and Claims for Beef, Chicken, and Pork.

*Total* *(N = 753,368)*		Beef (N = 425,761)			Chicken (N = 196,450)			Pork (N = 131,157)		
*Product Claim*		Average Price ($/lb)	Frequency	Percent	Average Price ($/lb)	Frequency	Percent	Average Price ($/lb)	Frequency	Percent
*Organic*	0	9.55	418,669	98.33	7.75	184,672	94.00	6.32	130,880	99.79
	1	15.93	7,092	1.67	8.31	11,778	6.00	11.51	277	0.21
*Grass-fed*	0	9.42	405,360	95.21	7.78	196,355	99.95	6.33	131,134	99.98
	1	14.34	20,401	4.79	10.19	95	0.05	9.32	23	0.02
*Gluten-free*	0	9.80	391,329	91.91	7.68	179,514	91.38	6.27	121,342	92.52
	1	7.97	34,432	8.09	8.86	16,936	8.62	7.08	9,815	7.48
*Non-GMO*	0	9.64	423,915	99.57	7.77	193,442	98.47	6.32	130,912	99.81
	1	14.10	1,846	0.43	8.94	3,008	1.53	12.67	245	0.19
*Less greenhouse gas*	0	9.65	424,291	99.65	7.78	196,216	99.88	–	–	–
	1	12.36	1,470	0.35	11.86	234	0.12	–	–	–

Given the national representation of major grocery chains, the summary statistics of the dataset provide several insights into the retail market availability of meat products with various claims. Overall, among the sustainability labels, the *Less greenhouse gas* label is a novelty across the market, representing a relatively small proportion of available beef (0.35%) and chicken products (0.12%). Beef has slightly more products with “less greenhouse gas” than chicken and this may possibly be because cattle produce much more GHG than chicken and hogs which is a big concern for cattle [US 51]. The *Less greenhouse gas* label is entirely absent from the pork products in the data, indicating these products are wholly unavailable at this point or are a particular rarity. This is not entirely surprising, however, given that specialty claims in pork products are, on the whole, less common than their counterparts in chicken and beef products. For example, while 6% of the chicken products in the sample carried an organic claim, less than 1% of the pork products did. Although the literature has well-established the existence of considerable consumer demand for these traits in meat products, the data indicate that the retail market has somewhat limited offerings of labeled products, an outcome perhaps more heavily influenced by supply-side constraints than demand opportunities.

Many prior studies might address why beef is the meat about which we see or hear the most concern about in its impact on environment; retailers provide *Less greenhouse gas* labels more on beef than other meats. According to the global meta-analysis of livestock [52], beef production generates around 5–10 times more GHG emissions per kg than chicken or pork primarily driven by more sources of emission such as enteric methane, land use change, and feed. Meanwhile, consumers are concerned more about beef than chicken and pork when it comes to environmental sustainability of meat production practices. Harmann and Siegrist [53] found that consumers rate beef as the least environmentally sustainable meat and chicken as a relatively climate-friendly meat. They further discuss that beef is the only meat that environmental concern reduces consumption [53]. Camilleri et al. [54] support that consumers prioritize beef when they are asked about climate-friendly diet shifts. Leiserwitz et al. [55] found from the U.S. survey that 57% of Americans think reducing beef consumption helps mitigate climate change, while only 19% think that reducing chicken consumption helps and pork is not mentioned at all. These findings help explain why retailers offer more *Less greenhouse gas* labels for beef than for other meat items.

### 3.2. Model results and discussion

In general, the model results illustrate that the coefficient signs for *Organic, Grass-fed, Gluten-free, Non-GMO,* and *Less greenhouse gas* labels are positive for beef, chicken, and pork products, which is consistent with economic intuition. Table 5 shows the results of the hedonic pricing model for online beef product offerings. The results generally satisfy our expectations in terms of coefficient sign of various labels’ presence and the related impacts on the product price. The coefficients for *Less greenhouse gas, Organic, Grass-fed, Gluten-free,* and *Non-GMO* labels were all statistically significant. The claim with the highest average premium was *Less greenhouse gas*. All coefficients were significant and the estimated coefficient for every label studied reflected a premium with the exception of *Gluten-free*, which was associated with a discount.

**Table 5:** Hedonic pricing model results: beef products.

Variable	Coefficient	Price Premium (%)
*Product Claim*		
Organic	0.282***	32.57
	(0.006)	
Grass-Fed	0.285***	32.97
	(0.003)	
Gluten-Free	−0.141***	−14
	(0.002)	
Non-GMO	0.220***	24.60
	(0.008)	
Less greenhouse gas	0.523***	68.70
	(0.005)	
*Region*		
Delta	−0.00866*	−0.87
	(0.004)	
Northeast	0.0544***	5.59
	(0.002)	
Southeast	0.0681***	7.04
	(0.003)	
Southern Plains	−0.0211***	−2.09
	(0.003)	
West	0.123***	13.08
	(0.002)	
*Store Chain*		
Albertsons	0.191***	21.04
	(0.003)	
Kroger	0.104***	10.96
	(0.003)	
*Cut*		
Ground beef	−0.242***	−22
	(0.002)	
Steak	0.565***	75.94
	(0.002)	
*Other*		
Promotion	−0.132***	−12.37
	(0.002)	
Constant	1.943***	
	(0.002)	
Observations	425,761	
R-squared	0.257	

Robust standard errors in parentheses; *** p < 0.01, ** p < 0.05, * p < 0.1. One (*), two (**), and three (***) asterisks denote coefficients significantly different from zero at the 0.10, 0.05, and 0.01 levels, respectively. Robust standard errors are in parentheses under the coefficient estimates.

Table 5 further presents results of price premiums estimated for beef with different claims over conventional beef. The *Less greenhouse gas* product claim had the highest estimated premium of 68.70% compared to conventional beef. However, the estimate being based on such a small number of observations, only 0.35% of the dataset, should not be ignored; impacts of this estimate should be interpreted with caution given how small of a share of the dataset made these claims. Direct hedonic estimates of price premiums for low-GHG beef in retail markets are scarce in the published literature, limiting direct comparison with prior studies. By comparison, we found that compared to conventional beef the coefficient for the *Organic* label was positive and significant, indicating an average premium of approximately 32.57%. *Grass-fed* product claims were associated with a premium of 32.97% and *Non-GMO* product claims with a premium of 24.60% relative to conventional beef. The coefficient of the *Gluten-free* products was significant but indicated a discount of approximately 14%. The average discount for a beef product being offered on a promotion was 12.37%. All regional coefficients were statistically significant at the 1% level, indicating significant beef price variation across major U.S. regions. Beef products offered in the West were the most expensive on average. The price in the Delta region and in the Southern Plains, relative to the Midwest was lower by 0.87% and 2.09% respectively, and in the Northeast, Southeast, and West was higher by 5.59%, 7.04%, and 13.08% respectively when compared to the Midwest area. The firms Albertsons and Kroger had higher prices by 21.04% and by 10.96% in comparison to the base firm, Meijer, without discounts considered. Compared to all beef cuts other than ground or steak, ground beef was associated with a discount of 22% but steak cuts carried an average premium of 75.94%. Overall, the results are in line with economic intuition about premiums. Certified products such as organic, and grass-fed are, in general, more expensive than their conventional counterparts. These findings are consistent with previous research, which showed positive premiums for environmentally friendly and sustainable attributes in beef and its associated products [20,21,44].

Results from the hedonic pricing model on chicken products (Table 6) show that, similar to beef, observed labels on products for *Organic, Grass-fed, Gluten-free, Non-GMO*, and *Less greenhouse gas* were statistically significant explanatory variables of price in comparison to conventional chicken without any of such labels. Also similar to beef, the *Less greenhouse gas* claim was associated with the highest premium of all claims. All coefficients were significant and the estimated coefficient for almost every label studied reflected a premium. The *Less greenhouse gas* products had a significant, positive premium 64.70%. As with beef, chicken products with this label represent only 0.12% of the dataset and this estimate should be interpreted with caution. As is the case of the *Less greenhouse gas* labeled beef products, direct hedonic estimates for low-GHG chicken are scarce in the literature. The coefficient for the *Organic* label on chicken was positive and significant, with an average premium of 7.29%. The *Grass-fed* products were associated with a positive premium of 16.88% and the *Non-GMO* products with a premium of 7.15%. Unlike the discount we observed for *Gluten-free* claims on beef products, the *Gluten-free* chicken products exhibited an average premium of 20.68%. Chicken products offered on a promotion had an average discount of 14.02%. The average price for chicken in the Delta region and in the Southern Plains was 4.61% and 7.41% lower than in the Midwest, respectively. In the Northeast region the price was higher by 1.34%, in the Southeast region was higher by 5.50%, and in the West was higher by 7.50%. Albertsons and Kroger had higher prices by 5.66% and by 10.05% compared to Meijer. Compared to all other chicken cuts such as whole chicken and/or others, chicken breast cuts were observed with a discount of 0.52% and leg, thigh, and wing cuts had an average discount of 19.51%.

**Table 6:** Hedonic pricing model results: chicken products.

Variable	Coefficient	Price Premium (%)
*Product Claim*		
Organic	0.070***	7.29
	(0.004)	
Grass-Fed	0.156***	16.88
	(0.018)	
Gluten-Free	0.188***	20.68
	(0.003)	
Non-GMO	0.069***	7.15
	(0.007)	
Less greenhouse gas	0.499***	64.70
	(0.007)	
*Region*		
Delta	−0.047***	−4.61
	(0.007)	
Northeast	0.013***	1.34
	(0.003)	
Southeast	0.054***	5.50
	(0.005)	
Southern Plains	−0.077***	−7.41
	(0.005)	
West	0.072***	7.50
	(0.003)	
*Store Chain*		
Albertsons	0.055***	5.66
	(0.005)	
Kroger	0.096***	10.05
	(0.005)	
*Cut*		
Chicken breast	−0.005***	−0.52
	(0.002)	
Chicken Leg/thigh/wing	−0.217***	−19.51
	(0.003)	
*Other*		
Promotion	−0.151***	−14.02
	(0.002)	
Constant	1.966***	
	(0.004)	
Observations	196,450	
R-squared	0.085	

Robust standard errors in parentheses; *** p < 0.01, ** p < 0.05, * p < 0.1. One (*), two (**), and three (***) asterisks denote coefficients significantly different from zero at the 0.10, 0.05, and 0.01 levels, respectively. Robust standard errors are in parentheses under the coefficient estimates.

Table 7 shows the results of the hedonic pricing model for pork products, along with the values of premiums or relative discounts for pork products. In this model (Table 7), *Organic, Grass-fed, Gluten-free,* and *Non-GMO* labels were statistically significant explanatory variables of product price and reflected a premium over conventional pork products without such specialty claims. The claim with the highest associated premium, 98.57%, was *Non-GMO*. However, this estimate should be interpreted with caution, as such products represent only 0.19% of the dataset. Impacts of this estimate should be interpreted with caution given how small of a share of the dataset made these claims. To our knowledge, no prior hedonic study has directly estimated the price premium for non-GMO pork in retail markets, limiting direct comparison with prior studies for validation. This differs from the models for chicken and beef products, where the highest premiums were associated with the *Less greenhouse gas* label, which is absent from the pork products currently available on the market. The *Organic* label had an average premium of 43.33%, the *Grass-fed* products were associated with a premium of 62.90%, and *Gluten-free* products had an average premium of 6.39%. When offered on a promotion, the average discount for pork products was 9.88%. Compared to the Midwest, the prices in the Northeast, Southeast, and West regions were higher by 1.90%, 5.12%, and 4.34%, respectively. In the Delta and Southern Plains regions, the average price was lower than in the Midwest by 2.89% and 9.3%, respectively. Albertsons and Kroger had higher prices by 24.23% and by 23.36% in comparison to Meijer. Compared to all other pork cuts, ham and bacon were observed to have premiums of 12.18% and 24.98% respectively.

**Table 7:** Hedonic pricing model results: pork products.

Variable	Coefficient	Price Premium (%)
*Product Claim*		
Organic	0.360***	43.33
	(0.034)	
Grass-Fed	0.488***	62.90
	(0.003)	
Gluten-Free	0.0620***	6.39
	(0.005)	
Non-GMO	0.686***	98.57
	(0.008)	
*Region*		
Delta	−0.0293***	−2.89
	(0.007)	
Northeast	0.0189***	1.90
	(0.004)	
Southeast	0.0500***	5.12
	(0.006)	
Southern Plains	−0.0976***	−9.30
	(0.006)	
West	0.0425***	4.34
	(0.004)	
*Store Chain*		
Albertsons	0.217***	24.32
	(0.005)	
Kroger	0.210***	23.36
	(0.005)	
*Cut*		
Ham	0.115*	12.18
	(0.060)	
Bacon	0.223***	24.98
	(0.007)	
*Other*		
Promotion	−0.104***	−9.88
	(0.003)	
Constant	1.591***	
	(0.003)	
Observations	131,157	
R-squared	0.048	

Robust standard errors in parentheses; *** p < 0.01, ** p < 0.05, * p < 0.1. One (*), two (**), and three (***) asterisks denote coefficients significantly different from zero at the 0.10, 0.05, and 0.01 levels, respectively. Robust standard errors are in parentheses under the coefficient estimates.

Overall, the results generally found premiums on meat products with specialty claims. Previous studies have also shown positive premiums and demand for products with sustainability labels. Van Loo et al., [2] found that Belgian consumers will pay a higher premium for free-range chicken breasts followed by animal welfare labeling, carbon footprint labels (30% and 20% CO2-reduction), and organic logos. Bir et al. [56] found positive willingness to pay for whole turkey attributes, including raised free range, fed a vegetarian diet, and prohibiting use of hormones and antibiotics. Byrd et al. [57] identified a positive willingness to pay for verified local production of chicken breasts, but not pork chops, highlighting the potential for product-specific valuation on specialty labeled products by consumers. Gschwandtner et al. [58] found an average organic premium of 135% for chicken in the United Kingdom. Thus, the results are consistent with earlier findings in the literature.

Moreover, the *Less greenhouse gas* label had the highest premiums for those products for which they were available in the market, beef and chicken. Results indicate that the *Less greenhouse gas* label can increase the average price of a meat product by approximately two-thirds, though it is unclear what proportion of that premium might be attributable to increased production costs versus rents for increased consumer value. As noted above, however, the higher premium for products with *Less greenhouse gas* may be due to the small sample size in the data. Apart from the *Less greenhouse gas* claim having the highest premium for both chicken and beef products, there is very little consistency observed in the relative value of each claim across the different meat types. For example, while *Non-GMO* had the highest premium of all claims in pork products, nearly doubling the price of a product relative to conventional, it was the least valuable of the claims studied for chicken products. Again, the small number of Non-GMO pork products in the dataset (0.19%) necessitates caution when interpreting premiums derived from such limited samples. These findings indicate that it may be difficult to predict the size of retail market premiums possible for a claim on a meat product based on the premiums garnered for a similar claim on a different type of meat. Nevertheless, with the exception of the *Gluten-free* claim in beef products, every claim evaluated carried a significant premium, indicating that the retail market rewards the underlying specialty practices, if with varying magnitude.

With the exception of *Less greenhouse gas*, which was absent from pork products, and *Gluten-free,* the premiums for the labels were highest for pork products. Premiums are both a reflection of increased consumer demand or value and increased costs of production, and specialty pork production can result in significantly higher costs of production than the highly efficient conventional standard of pork production in the United States [59]. Thus, the observation of the highest premiums on pork products for *Organic, Non-GMO,* and *Grass-fed* is not surprising. In contrast, the premiums for these claims were consistently lowest in chicken products. The only claim which did not follow this pattern across the three meat types was *Gluten-free*, for which chicken products bore the highest premium and beef products received a discount. This indicates that, while *Gluten-free* is similar in nature to the other claims in announcing the absence of an unwanted substance or practice, there is evidence of a clear distinction in the market for absence claims related to post-farm gate ingredients that might be present in a finished product.

Although the presence of specialty claims tended to have the highest magnitude of influence on price variation, the results also demonstrate that meat product prices are significantly and consistently affected by region of sale, store chain, and cut or product type. When it comes to geographical variations in prices, products offered in the Delta and Southern Plains regions were cheaper than the Midwest, while products in the Northeast, Southeast, and West were more expensive than the Midwest. Compared to Meijer, products in Albertsons and Kroger were more expensive. Unsurprisingly, ground beef was cheaper and steak was more expensive for beef, while ham and bacon were more expensive than other pork products. These findings are consistent with expectations as well as with the general findings in the literature [44].

## 4. Conclusion

Consumers have become more aware of carbon footprints and environmental implications of differing production systems. In particular, interest in the carbon footprints of various production systems and for various meat products has increased although the markets for products with less greenhouse gas emissions is still a very small niche market. Meat products with sustainability labels have become more prevalent as consumers’ interest in various forms of agricultural production has increased alongside growing concerns about animal welfare, food safety, economic viability, and sustainability of farming and communities [60]. Indeed, these elements might be seen as adding value to the quality of animal-derived products [21].

In this study, we examined the price premium for sustainability labels such as organic, less greenhouse gas, grass-fed, gluten-free, and non-GMO by utilizing data web-scraped from grocery websites. Beef, chicken, and pork products in selected stores were studied with regard to labels in order to identify the availability of specialty products, as well as the premiums associated with various claims. For beef and chicken, among the evaluated claims, the *Less greenhouse gas* claim had the highest premium. There were no pork products identified in the market with *Less greenhouse gas* claims. Given the premiums observed for beef and chicken products with this claim, it is reasonable to expect that pork products produced with lower GHG emissions could garner a premium price in the retail market, representing an opportunity for pork producers. However, the substantial variation in premiums for other claims across meat types makes it unclear to what extent premiums for sustainability labels on pork products might compare in magnitude to those of beef and chicken products. As expected, sustainability labeled as *organic*, less greenhouse gas, *grass-fed*, *Gluten-free, and Non-GMO* were, in general, more expensive than their conventional products for all meat products, with the only identified instance of a discount being for *Gluten-free* claims on beef products. Altogether, our results indicate that retail market value for claims in meat products persists across the United States, with high premiums exhibited for *Less greenhouse gas* labels despite a relatively low prevalence of these products for beef and chicken and their complete market absence for pork [11,45,61].

A limitation of this analysis is the cross-sectional nature of the data collected. Given seasonal variation in demand for meat products, a longer period of study may elicit different findings with respect to premiums on various cuts of meat. Future analysis of interest in livestock products with sustainability labels can be extended to a time-series study so that changes can be compared and contrasted over time, which would then allow for studying seasonality in meat promotions and marketing campaigns. Research comparing consumers’ interest and behavior and the environmental policies among various countries could also be informative, as it could examine whether the findings hold in differing countries with differing cultures, production systems, and social norms.

## Supporting information

S1 Data Dataset collected from the grocery stores by web-scraping. (CSV)
